# Psychological Disorders and Psychosocial Resources of Patients with Newly Diagnosed Bladder and Kidney Cancer: A Cross-Sectional Study

**DOI:** 10.1371/journal.pone.0155607

**Published:** 2016-05-18

**Authors:** Yi-Long Yang, Li Liu, Meng-Yao Li, Meng Shi, Lie Wang

**Affiliations:** 1 Department of Social Medicine, School of Public Health, China Medical University, Shenyang, Liaoning, PR China; 2 Department of English, School of Basic Medicine, China Medical University, Shenyang, Liaoning, PR China; Max-Planck-Institute for Experimental Psychiatry, GERMANY

## Abstract

**Purpose:**

Psychological disorders have been proven to be associated with poor physiological, psychological and immune outcomes in cancer patients. However, despite of many challenges of the changed self-image/body image and the altered sexual/urinary function, relatively little is known about psychological disorders of patients with newly diagnosed bladder and kidney cancer. We aimed to investigate the prevalence of depression, anxiety, post-traumatic stress disorder (PTSD) and the associated psychosocial factors among bladder/kidney cancer patients.

**Methods:**

A cross-sectional study was conducted of consecutive inpatients with bladder/kidney cancer in the First Affiliated Hospital of China Medical University in Liaoning Province, northeast China. A total of 489 early-stage cancer patients eligible for this study completed questionnaires on demographic and clinical variables, depression, anxiety, PTSD, perceived social support and positive psychological variables (hope, optimism and resilience) anonymously during October 2013 and August 2014. Hierarchical regression analysis was used to examine the relationships between psychosocial resources and psychological disorders, while controlling for possible covariates.

**Results:**

The prevalence of depression, anxiety and PTSD was 77.5%, 69.3% and 25.2%, respectively, while 24.9% of patients had psychological co-morbidity. Psychosocial resources together explained more than one-third of the variance on psychological disorders. Under standardized estimate (β) sequence, patient’s perception of social support from family was significantly associated with depression, anxiety and PTSD (*p* < 0.01). Optimism and resilience showed integrated and independent effects on psychological disorders, and hope represented the significant association with PTSD only (*p* < 0.01).

**Conclusions:**

The high prevalence of psychological disorders in newly diagnosed patients with early-stage bladder/kidney cancer should receive more attention in Chinese medical settings. Additionally, in consideration of the different protective effects of psychosocial resources, the present study demonstrated that one complete psychological intervention integrating the associated psychosocial factors are necessary to ameliorate psychological disorders so as to provide patients with a more holistic cancer care.

## Introduction

Bladder cancer and kidney cancer are common tumors of urinary systemin China [1]. Compared to other malignant tumors, the incidence rate and mortality rate of bladder/kidney cancer was relatively low [2], and bladder (67.3%) and kidney cancer (62.0%) had high level of age-standardized 5-year relative survival [3]. Consequently, there has been an increasing interest with regards to the evaluation of quality of life in bladder/kidney cancer patients [4–6], such as long-term survivors, but not much is generally known regarding the mental health issues of newly diagnosed patients.

In reality, bladder/kidney cancer patients were faced with multiple stressors, including pain, fatigue, significant body changes and the altered sexual/urinary function [7,8]. Besides, intense physiological, psychological and interpersonal challenges might emerge the first year after the cancer diagnosis. All these unique characteristics might cause and aggravate patients’ psychological disorders, such as depression, anxiety and post-traumatic stress disorder (PTSD), which are the most common psychological issues encountered with cancer patients [9,10]. However, there were very few studies on the evaluation of depression, anxiety and PTSD among bladder/kidney cancer patients [9,11,12]. More importantly, these psychological disorders could lead to impairment of immune response, prolonged recovery times, difficulty with symptom control, poor compliance with treatment and possibly reduced survival time [10,13,14]. Therefore, regularly screening and properly managing these psychological disorders is an important aspect of clinical oncology in these cancer patients.

Unsurprisingly, it is of great importance to identify the associated factors for psychological disorders in cancer patients, and psychosocial factors attracted great clinical attention because they could be modified through appropriate psychosocial inventions [15,16]. Although a variety of psychosocial factors have been identified as predictors of depression, anxiety and PTSD in patients diagnosed with different kinds of cancer, the unique psychological issues in bladder/kidney cancer patients might create substantial obstacles to develop effective prevention for psychological disorders. Thus, the associated protective psychosocial factors should be identified in order to develop effective strategies for depression, anxiety and PTSD.

Psychosocial factors associated with psychological disorders might be roughly divided into external and internal factors. Among external factors, research has generally shown social support to be beneficial in cancer patients, which has been extensively demonstrated to improve psychological as well as physical well-being [17,18], and to act as a buffer against stressful life events [19]. In contrast, social isolation and inadequate social support leaded to hopelessness, relational strain and impaired communication [20–22]. However, studies neither assessed the association between different sources of social support and psychological disorders nor clearly distinguished which sources of social support was the essential resource for combating psychological disorders in cancer patients.

On the other hand, positive psychological factors have received increasing attention in oncology under influence of positive psychology and psycho-oncology. Our previous study has assessed the integrated effects of hope, optimism and general self-efficacy on depression/anxiety in cervical cancer patients [23], but the results were limited given the sample size (N = 224) and gender. The present study seeks to partly replicate these results and further extend previous research by analyzing the associations between Snyder’s theory of hope, resilience, optimism and psychological disorders in newly diagnosed bladder/kidney cancer patients concerning early-middle stage and gender that has relatively good representation (N > 400).

Hope is conceptualized as goal-directed thinking and positive motivational state that consists two parts: agency (one’s perception that he or she can sustain and persevere toward achieving goals) and pathways (the perceived ability to find methods or routs of reaching the desired goals) [24]. Hope is an important factor in the lives of cancer patients, including helping to adjust to cancer and reducing psychological distress [25]. Optimism also appears to be an important predictor of psychological disorders among cancer patients [26]. It is a relatively stable tendency to expect that good rather than bad things will happen, and the general positive outcome expectation can enhance the continuous efforts in achieving the given goal [27]. Compared to the constructs of hope and optimism, resilience has been more frequently studied among the people faced with traumatic events. Resilience is expressed in an individual’s capacity to successfully recover and maintain their mental health in the context of traumatic events [28], and patients’ resilience could influence the status of psychosocial adaptation in response to cancer [29].

Hope, optimism and resilience have been proven to be beneficial in cancer patients [24–29], and each construct represented different aspects of positive psychological resource. However, there are few studies particularly exploring their integrated and independent effects on psychological disorders when assessed together, and perceived social support and positive psychological resources were also seldom integrated into a holistic cancer care to combat depression, anxiety and PTSD. The objectives of the study were to identify the prevalence of depression, anxiety and PTSD in newly diagnosed bladder/kidney cancer patients, and to examine their associated psychosocial factors after adjusting for the demographic and clinical variables. Particularly, the present study further explored the different sources of social support and distinguished between different effects of hope, optimism and resilience on psychological disorders.

## Materials and Methods

### Study design and sample

A cross-sectional study was conducted of consecutive inpatients with bladder and kidney cancer during October 2013 and August 2014. The study took place in Department of Urology in the First Affiliated Hospital of China Medical University, which is an important provider of cancer services to a geographically defined area of 8.2 million people in the south of northeastern China. The eligibility criteria for patient recruitment were (1) 18 ≤ age < 80 years, (2) being histologically diagnosed with bladder and kidney cancer, (3) aware of the cancer diagnosis, (4) able to understand and read Chinese well enough to answer the questionnaires, (5) newly diagnosed patients (time since diagnosis ≤ 12 months). Exclusion criteria were the following: (1) patients had a history of psychiatric problems before cancer diagnose, (2) patients had intellectual and/or cognitive impairments, (3) patients had other active cancers. All registered patients’ attending physicians discussed eligibility on a case-by-case basis to avoid biased judgment and selection bias. After obtaining written consent, clinical data were collected from the medical record, and a set of self-report questionnaires was distributed to patients at the time of hospitalization.

The Committee on Human Experimentation of China Medical University reviewed and provided the ethics approval for this study, and the study procedures were in accordance with the ethical standards. All of the participants provided written informed consent after being orally informed about the study protocol, and they were totally voluntary and anonymous. We protected the privacy of patients in processing personal data and maintained confidentiality of individual records and accounts. The participation in this study did not affect the future free health examination and treatment which is standard in China.

Among a total of 595 registered patients, 5 patients had other active cancers (colon cancer, prostatic cancer and neuroendocrine cancer), and there were 29 patients older than 79 years. Additionally, 12 patients were not newly diagnosed cancers. Of 549 eligible patients for this study, 60 were excluded from analysis (> 30% missing data). Finally, we received effective responses from 489 bladder and kidney cancer patients with effective response rate 89.1%.

### Measurement of depression

Center for Epidemiologic Studies Depression Scale (CES-D) is a 20-item measure of the severity of depressive symptoms [30]. Items are ranked on a four-point frequency scale from 0 (never) to 3 (always). Higher scores reflect worse depressive symptoms, and a score ≥16 indicates probable clinical depression [30,31]. The CES-D has been shown to have good reliability and validity in cancer patients [22,31]. In this study, the Cronbach’s alpha for CES-D was 0.871.

### Measurement of anxiety

Zung Self-Rating Anxiety Scale (SAS) is designed to describe the presence and quantify the severity of anxiety as a clinical disorder [32]. SAS contains 20 items rated on a four-level Likert scale from 1 (not at all) to 4 (most of the time). The raw score should be standardized (raw score*1.25), and a score of 50 (raw score is 40) or higher represents presence of anxiety symptoms [32,33]. SAS was well validated and commonly used in cancer patients, including Chinese population [33,34]. In this study, the Cronbach’s alpha for SAS was 0.850.

### Measurement of PTSD

The PTSD Checklist-Civilian Version (PCL-C) assessed the PTSD symptoms criteria, including intrusion, avoidance, and hyperarousal according to the DSM-IV [35]. PCL-C is a 17-item self-rating questionnaire using a five-point Likert scale ranging from 1 (not at all) to 5 (extremely). The PCL-C yields a total score (17 to 85) with higher scores indicative of more PTSD symptoms. A total score of 50 or more suggests probable diagnosis of PTSD [36,37]. The term of ‘distressing event’ was replaced by the term of ‘cancer or cancer treatment’. The PCL-C has shown good validity and reliability in cancer patients [17,36,37]. In this study, the Cronbach’s alpha for PCL-C, intrusion, avoidance, and hyperarousal subscale was 0.953, 0.886, 0.901, and 0.920, respectively.

### Measurement of perceived social support

Multidimensional Scale of Perceived Social Support (MSPSS) [38] is 12-item measure of the sources of perceived social support, rated on a seven-point scale from 1 (very strongly disagree) to 7 (very strongly agree). It provides a summary score (12 to 84), as well as three subscales for perceived support from family, friends, and significant others. The MSPSS was validated and commonly used in cancer patients [18,39]. In this study, the Cronbach’s alpha was 0.969, 0.933, 0.917, and 0.928 respectively for MSPSS, family, friends, and significant others subscales.

### Measurement of hope

Adult Hope Scale (AHS) including eight items and four filler items rated on 4-point scales (1 = strongly disagree, 4 = strongly agree) [24]. The AHS contains four Agency and four Pathways items, and a high score denotes a higher level of pathways and agency. The hope level is the sum of the agency and pathways items. The AHS and its Chinese version have been used in cancer patients with acceptable validity and reliability [40]. The Cronbach’s alpha was 0.756, 0.731, and 0.838 respectively for the pathway, agency and AHS.

### Measurement of optimism

The Life Orientation Scale-Revised (LOT-R) was used to assess optimism [26,27], including 6 items (3 positively worded and 3 negatively worded items) and 4 filler items. The responses for each range from 1 (strongly disagree) to 5 (strongly agree). Because several studies demonstrated bidimensionality (optimism and pessimism) rather than unidimensionality for the LOT-R [26], LOT-R Optimism (total of the 3 positively worded items) was calculated as the indicator of optimism in this study. The Cronbach’s alpha was 0.616 for LOT-R Optimism.

### Measurement of resilience

The 14-items version of Resilience Scale (RS-14), a short version of the original RS (i.e. RS-25), was used to assess resilience [41]. RS-14 consists of 14 items rated on a 7-point scale, ranging from 1 (strongly disagree) to 7 (strongly agree). The total score ranges from 14 to 98 scores, with higher scores indicating higher resilience. The Chinese version of RS-14 had good validity and reliability among cancer patients [42]. In this study, the Cronbach’s alpha was 0.947 for RS-14.

### Demographic and clinical characteristics

The demographic variables included age, gender, marital status and education. Caner type, caner stage, time since diagnosis (months), treatment, and metastasis were included as the clinical variables.

### Statistical analysis

The Statistical Package for the Social Sciences (SPSS, version 13.0) was used to perform the statistical analyses, with two-tailed probability value of < 0.05 considered to be statistically significant. Inspection of histograms and analysis of skewness and kurtosis values for study variables revealed that data were approximately normally distributed.

Descriptive statistics of the demographic and clinical variables were indicated with median, mean, standard deviation (SD), number (N) and percentage (%) as appropriate. The distributions of CES-D, SAS and PCL-C in categorical variables were calculated using independent sample *t*-test and one way analysis of variance (ANOVA). When one-way ANOVA was found to be significant, least-significant-difference (LSD) was done to perform multiple comparisons. Pearson’s correlation was used to examine correlations among psychosocial variables.

Hierarchical regression analysis was used to explore the relationship between psychosocial resources and psychological disorders with adjustment for demographics and clinical variables related to psychological disorders in univariate analysis (*p* < 0.05). In Step 1, age, gender, education, time since diagnosis and cancer stage were added. There were two models (Model 1 and Model 2) in Step 2. Total score of MSPSS was added in Model 1, and three subscales of MSPSS (e.g., others, friend and family) were added in Model 2. Due to the high correlations among the MSPSS subscales, these variables were adjusted in the stepwise regression in Step 2 (Model 2). Hope, optimism and resilience were added in Step 3.We provided data including R^2^, adjusted R^2^ (Adj.R^2^), R^2^-changes, F, standardized regression coefficient (β) and *p* value for each step in the regression model. Moreover, tolerance (> 0.10) and variance inflation factor (< 5) were used to check for multicollinearity.

## Results

Table 1 presented patients characteristics. The patients (N = 489) were in the age range of 18–79 (Mean ± SD: 59.10 ± 11.28). Approximately 90% of the patients were married or living with a partner, and 48.7% received middle school education. The mean number of months after diagnosis was 6.04 (range: 1–12). Majority of patients (58.3%) were diagnosed at cancer stage I, and 99.4% were free of metastases. The prevalence of depression, anxiety and PTSD in bladder/kidney cancer patients was 77.5%, 69.3% and 25.2%, and 24.9% (N = 122) of patients had psychological co-morbidity (i.e., triad symptoms of depression, anxiety and PTSD).

**Table 1:** Patient Characteristics (N = 489).

	N	%	Mean (SD)	Median (Range)
**Demographic variables**				
Age (years)			59.10(11.28)	60.00(18–79)
≤ 55	170	34.8		
56–65	164	33.5		
≥ 66	155	31.7		
Gender				
Male	345	70.6		
Female	144	29.4		
Marital status				
Married/ cohabitation	437	89.4		
Single/widowed/divorced	52	10.6		
Education				
Middle school or below	238	48.7		
High school	126	25.8		
Junior college or above	125	25.6		
**Clinical variables**				
Cancer type				
Bladder cancer	244	49.9		
Kidney cancer	245	50.1		
Time since diagnosis (months)			6.04 (3.55)	5.00(1–12)
≤ 3	149	30.5		
4–6	143	29.2		
7–12	197	40.3		
Cancer stage				
I	285	58.3		
II	204	41.7		
Treatment type				
No treatment	4	0.8		
Surgery	294	51.9		
Chemotherapy	1	0.2		
Combined treatment	230	47.0		
Metastasis				
No	486	99.4		
Yes	3	0.6		
Recurrent				
Yes	14	2.9		
No	475	97.1		
CES-D (Scores ≥ 16)	379	77.5		
SAS (Scores ≥ 50)^a^	339	69.3		
PCL-C (Scores ≥ 50)	123	25.2		
Psychological co-morbidity^b^	122	24.9		

CES-D = Center for Epidemiologic Studies Depression Scale; SAS = Zung Self-Rating Anxiety Scale; PCL-C = Posttraumatic Stress Disorder Checklist-Civilian Version.

^a^ Standard score was used.

^b^ Patients suffered from both depression, anxiety and PTSD.

Table 2 revealed the depression, anxiety and PTSD scores in demographic and clinical variables. Education, cancer stage and time since diagnosis were significantly associated with depression, anxiety and PTSD (*p* < 0.05). Table A in S1 Appendix also indicated the association between psychosocial resources and cancer type. The level of social support form friends was higher in kidney cancer patients than that of bladder cancer patients. The intercorrelations between study variables were shown in Table 3. Perceived social support and positive psychological resources were negatively associated with depression, anxiety and PTSD (r = ranged from -0.361 to-0.481; *p* < 0.01).

**Table 2:** CES-D, SAS, PCL-C scores in demographic and clinical variables.

	CES-D	*p*-value	SAS	*p*-value	PCL-C	*p*-value
**Demographic variables**						
Age		0.806		0.586		0.307
≤ 55	23.17±9.63		43.98±9.15		37.42±13.99	
56–65	23.78±8.51		44.82±9.03		39.51±12.59	
≥ 66	23.20±9.96		43.81±9.94		37.72±13.21	
Gender		0.309		0.616		0.670
Male	23.11±9.42		44.07±9.38		38.05±13.43	
Female	24.05±9.22		44.54±9.35		38.61±12.98	
Marital status		0.534		0.925		0.583
Married/ cohabitation	23.29±9.32		44.22±9.32		38.11±13.45	
Single/widowed/divorced	24.15±9.77		44.09±9.81		39.09±11.91	
Education		0.001		<0.001		0.002
Middle school or below	23.93±9.26^a^		44.58±9.03^a^		38.95±12.83^a^	
High school	24.86±8.22^a^		46.22±8.29^a^		40.34±12.76^a^	
Junior college or above	20.85±10.19^b^		41.48±10.39^b^		34.68±14.08^b^	
**Clinical variables**						
Cancer type		0.766		0.506		0.359
Bladder cancer	23.26±9.41		43.93±9.63		38.77±13.29	
Kidney cancer	23.51±9.33		44.49±9.10		37.66±13.29	
Time since diagnosis		0.014		0.010		0.001
≤ 3	25.09±8.36^a^		46.06±8.43^a^		41.61±12.33^a^	
4–6	23.34±9.01		43.90±9.87^b^		37.41±13.28^b^	
7–12	22.12±10.14^b^		43.03±9.48^b^		36.24±13.57^b^	
Treatment		0.366		0.803		0.795
Combined treatment	23.77±8.96		44.30±9.51		38.04±13.16	
Surgery	23.00±9.77		44.09±9.29		38.36±13.48	
Cancer stage		0.012		0.008		0.002
I	22.49±9.55		43.27±9.62		36.62±12.90	
II	24.64±8.96		45.51±8.84		40.44±13.53	
Recurrent		0.085		0.148		0.966
Yes	19.14±10.38		40.64±10.75		38.07±13.86	
No	23.51±9.32		44.32±9.32		38.23±13.29	

CES-D = Center for Epidemiologic Studies Depression Scale; SAS = Zung Self-Rating Anxiety Scale; PCL-C = Posttraumatic Stress Disorder Checklist-Civilian Version.

^a,b^ Calculated by least-significant-difference (LSD), mean scores for depression, anxiety and PTSD with unequal superscripts differ significantly at the *p* < 0.05 level.

Independent sample *t*-test and one-way ANOVA were used.

**Table 3:** Means, standard deviation, range and zero-order correlations (Pearson’s r) among study variables.

Variables	Mean ± SD	Test Range	Sample Range	1	2	3	4	5	6	7	8	9	10
**1. CES-D**	23.38±9.36	0–60	0–44	1	0.864**	0.782**	-0.408**	-0.393**	-0.411**	-0.361**	-0.430**	-0.481**	-0.479**
**2. SAS**	44.21±9.36	20–80	20–66		1	0.774**	-0.438**	-0.427**	-0.450**	-0.372**	-0.432**	-0.464**	-0.468**
**3. PCL-C**	38.22±13.29	17–85	17–75			1	-0.422**	-0.409**	-0.425**	-0.370**	-0.453**	-0.457**	-0.440**
**4. Total-MSPSS**	58.42±17.28	12–84	20–84				1	0.964**	0.963**	0.931**	0.481**	0.353**	0.506**
**5. MSPSS-others**	19.40±6.11	4–28	4–28					1	0.916**	0.838**	0.457**	0.370**	0.491**
**6. MSPSS-family**	20.12±6.27	4–28	6–28						1	0.833**	0.457**	0.348**	0.488**
**7. MSPSS-friend**	18.89±5.74	4–28	6–28							1	0.462**	0.288**	0.468**
**8. AHS**	21.64±4.45	8–32	8–32								1	0.443**	0.569**
**9. LOT-R Optimism**	10.09±2.07	3–15	5–15									1	0.376**
**10.RS-14**	64.89±17.11	14–98	14–98										1

CES-D = Center for Epidemiologic Studies Depression Scale; SAS = Zung Self-Rating Anxiety Scale; PCL-C = Posttraumatic Stress Disorder Checklist-Civilian Version; MSPSS = Multidimensional Scale of Perceived Social Support; AHS = Adult Hope Scale; LOT-R = Life Orientation Scale-Revised; RS-14 = 14-items version of Resilience Scale

** Correlation is significant at the 0.01 level (two-tailed).

Tables 4–6 indicated the final results of the hierarchical regression analyses of depression, anxiety and PTSD after adjusting for demographic and clinical variables. Psychosocial resources together accounted for an additional variance of depression (34.9%), anxiety (34.4%) and PTSD (33.3%). Perceived social support-family was the only external resources of depression (β = -0.143, *p* = 0.001), anxiety (β = -0.206, *p* < 0.001), and PTSD (β = -0.170, *p* < 0.001). Both optimism and resilience were significantly associated with depression, anxiety and PTSD, respectively, and hope showed significantly independent effect on PTSD (β = -0.153, *p* = 0.001) when they entered into regression together. Tolerance (range: 0.557–0.981) and variance inflation (range: 1.019–1.794) did not indicate an obvious multicollinearity problem.

**Table 4:** Hierarchical regression analyses for psychosocial resources predicting depression.

Variables	Step 1(β)	Step 2(β)		Step 3(β)	
		Model 1	Model 2	Model 1	Model 2
**Covariates**					
Age	0.014	0.006	0.018	0.036	0.040
Gender	0.038	0.048	0.049	0.048	0.049
Education1	0.145**	0.052	0.052	0.019	0.016
Education2	0.171**	0.105*	0.113*	0.027	0.029
Time since diagnosis	-0.123**	-0.053	-0.048	-0.020	-0.016
Cancer stage	0.076	0.048	0.045	0.038	0.036
**Predictors (external)**					
Perceived social support		-0.378***		-0.127**	
Perceived social support-others			-		-
Perceived social support-family			-0.384***		-0.143**
Perceived social support-friend			-		-
**Predictors (internal)**					
Hope				-0.077	-0.076
Optimism				-0.304***	-0.301***
Resilience				-0.244***	-0.241***
**F**	4.544***	15.389***	15.770***	27.116***	27.507***
**R**^**2**^	0.054	0.183	0.187	0.362	0.365
**Adj.R**^**2**^	0.042	0.171	0.175	0.349	0.352
**R**^**2**^**-changes**	0.054	0.129	0.133	0.179	0.179

β = standardized regression coefficient; Education1 = Middle school or below vs. Junior college or above; Education2 = High school vs. Junior college or above; Adj.R^2^ = adjusted R^2^.

* *p* < 0.05

** *p* < 0.01

*** *p* < 0.001

There were two models (Model 1 and Model 2) in Step 2. Total score of social support was added in Model 1, and three subscales (i.e., others, friend and family) were added in Model 2 adjusted by the stepwise regression due to the high correlations among the MSPSS subscales

**Table 5:** Hierarchical regression analyses for psychosocial resources predicting anxiety.

Variables	Step 1(β)	Step 2(β)		Step 3(β)	
		Model 1	Model 2	Model 1	Model 2
**Covariates**					
Age	-0.002	-0.010	0.003	0.014	0.020
Gender	0.016	0.027	0.029	0.027	0.029
Education1	0.149**	0.049	0.047	0.017	0.013
Education2	0.205***	0.134**	0.142**	0.065	0.067
Time since diagnosis	-0.130**	-0.055	-0.048	-0.025	-0.020
Cancer stage	0.078	0.048	0.045	0.040	0.037
**Predictors (external)**					
Perceived social support		-0.407***		-0.178***	
Perceived social support-others			-		-
Perceived social support-family			-0.422***		-0.206***
Perceived social support-friend			-		-
**Predictors (internal)**					
Hope				-0.089	-0.087
Optimism				-0.268***	-0.264***
Resilience				-0.209***	-0.203***
**F**	5.360***	18.512***	19.765***	26.543***	27.458***
**R**^**2**^	0.063	0.212	0.223	0.357	0.365
**Adj.R**^**2**^	0.051	0.201	0.212	0.344	0.352
**R**^**2**^**-changes**	0.063	0.150	0.161	0.145	0.141

β = standardized regression coefficient; Education1 = Middle school or below vs. Junior college or above; Education2 = High school vs. Junior college or above; Adj.R^2^ = adjusted R^2^.

** *p* < 0.01

*** *p* < 0.001

There were two models (Model 1 and Model 2) in Step 2. Total score of social support was added in Model 1, and three subscales (i.e., others, friend and family) were added in Model 2 adjusted by the stepwise regression due to the high correlations among the MSPSS subscales

**Table 6:** Hierarchical regression analyses for psychosocial resources predicting PTSD.

Variables	Step 1(β)	Step 2(β)		Step 3(β)	
		Model 1	Model 2	Model 1	Model 2
**Covariates**					
Age	0.029	0.021	0.033	0.041	0.046
Gender	0.012	0.021	0.023	0.022	0.023
Education1	0.142**	0.047	0.047	0.012	0.010
Education2	0.167**	0.100*	0.108*	0.029	0.032
Time since diagnosis	-0.150**	-0.079	-0.074	-0.050	-0.046
Cancer stage	0.098*	0.070	0.067	0.062	0.060
**Predictors (external)**					
Perceived social support		-0.384***		-0.154**	
Perceived social support-others			-		-
Perceived social support-family			-0.390***		-0.170***
Perceived social support-friend			-		-
**Predictors (internal)**					
Hope				-0.153**	-0.153**
Optimism				-0.263***	-0.260***
Resilience				-0.156**	-0.153**
**F**	5.718***	17.128***	17.532***	25.318***	25.759***
**R**^**2**^	0.066	0.200	0.203	0.346	0.350
**Adj.R**^**2**^	0.055	0.188	0.192	0.333	0.337
**R**^**2**^**-changes**	0.066	0.133	0.137	0.147	0.147

β = standardized regression coefficient; Education1 = Middle school or below vs. Junior college or above; Education2 = High school vs. Junior college or above; Adj.R^2^ = adjusted R^2^.

* *p* < 0.05

** *p* < 0.01

*** *p* < 0.001

There were two models (Model 1 and Model 2) in Step 2. Total score of social support was added in Model 1, and three subscales (i.e., others, friend and family) were added in Model 2 adjusted by the stepwise regression due to the high correlations among the MSPSS subscales

Additionally, the related analyses were conducted specifically with bladder cancer in our study. Tables B-D in S1 Appendix presented the study variables scores in clinical variables (i.e., cancer stage, surgical methods and recurrent), and Tables E-G in S1 Appendix indicated the association between psychosocial resources and psychological disorders in bladder cancer patients with stage I or stage II after adjusting for covariates.

## Discussion

This is the first comprehensive cross-sectional study to explore the prevalence and associated psychosocial resources of depression, anxiety and PTSD among bladder and kidney cancer patients.

Our previous studies found that depression and anxiety coexisted in Chinese cancer patients [9,23]. In the present study, approximately one-fourth of patients (24.9%) experienced psychological co-morbidity, indicating that depression, anxiety and PTSD also coexisted among bladder and kidney cancer patients. This is noteworthy because co-morbid psychological disorder tended to have severe symptoms, poorer outcomes and greater use of healthcare resources than those with a single disorder [43]. Additionally, the prevalence of depression-anxiety comorbidity in this study (68.5%) was higher than the results of our previous empirical study in cervical cancer patients (45.5%) [23].

The prevalence of depression, anxiety and PTSD was 77.5%, 69.3% and 25.2% in bladder and kidney cancer patients, respectively, which was higher than the findings of previous studies using the same scales and cut-off score among different kinds of cancer, including prostate cancer, breast cancer, and ovarian cancer [31,34,36,37,44,45]. There might be two reasons for the different prevalence. First, according to the previous literatures, patients with bladder/kidney cancer were mainly faced with the changed self-image/body image and the altered sexual/urinary function, which might have an adverse impact on their psychological well-beings. Besides, these patients might also face the physiological, psychological and interpersonal challenges across the first year after the cancer diagnosis. Second, cancer patients of the above studies [31,34,36,37,44,45] were mainly from developed countries which may have lower prevalence of psychological disorders as compared to developing countries like China [46]. Several studies indicated the high prevalence of depression and anxiety in Chinese cancer patients using a variety of self-report questionnaires [9,23,33]. Compared with the developed countries, clinical practice guidelines for the psychotherapy and supportive care of cancer patients are still not available in China [16]. Therefore, the above situations, combined with the psychological issues that received little attention, might aggravate psychological disorders in patients across the first year after diagnosed with bladder/kidney cancer.

The present study found that a relatively few patients were identified as likely to experience PTSD symptoms, although the prevalence of PTSD symptoms was higher than that in other studies using the same cut-off score (PTSD score ≥ 50) [36,37]. The low prevalence of PTSD (ranging from 3% to 35%) has been found in cancer patients [21], and our findings also seemed to support that although psychological disorder is the major issue and challenge in taking care of cancer patients, a definite portion of patients could successfully deal with cancer-related PTSD.

One of the core findings in this study was that psychosocial resources together explained more than one-third of the variance on psychological disorders, and perceived social support and positive psychological resources showed significant negative association with psychological disorders (*p* < 0.01). Most studies to date mainly focused on the independent effect of internal or external factors on reducing psychological disorders [17,18,26,28,40], and ignored, to some extent, the integrated effects of internal and external resources. The present scarcity in the study of psychological issues among bladder/kidney cancer patients provided such a suitable platform to investigate the relationship between psychosocial resources and psychological disorders.

We found that perceived social support (total score) was significantly and negatively associated with psychological disorders (*p* < 0.01), and this findings converged with results from other studies in cancer patients [18,22]. However, when different sources of social support were further explored, an interesting finding was that only social support from family was significantly associated with depression, anxiety and PTSD (*p* < 0.01), contrary to the general conclusion that friends and significant others were also primary sources of support for cancer patients [18,22]. We speculated that in Chinese society, people generally have stronger sense of belonging to a family, and family members can support each other emotionally and financially. Especially in the context of traumatic events, people feel responsible for looking after their relatives, and the care and concern of family members might be of great importance for patients. Additionally, psychosocial interventions involving family member have been proven to be beneficial for depression in cancer patients [47]. On the other hand, bladder and kidney cancer patients might not get enough supports from friends and significant others. Due to the adverse effects of changed self-image/body image and altered sexual/urinary function, patients might distance themselves from friends and others as they are afraid of losing affected person in their social network, and they do not know how to ask for help because of their embarrassment [39,48]. Friends and significant others might also avoid them because they feel awkward and hardly know what to do and say when they encounter these patients [48]. As a result, support from family members played an important or even the only role in ameliorating psychological disorders among bladder/kidney cancer patients.

Due to the association between cancer stage and treatment patterns specifically with bladder cancer, the analysis of patients with bladder cancer that have stage I or II was separated (Tables E-G in S1 Appendix). Perceived social support-family was the only external resources of psychological disorders among bladder cancer patients with stage I, while only social support from others was significantly associated with psychological disorders among patients with stage II. These differences might be ultimately attributed to the discrepancy in treatment patterns. Bladder cancer patients with stage I were mainly treated with transurethral resection of bladder tumor (TURBT), and patients with stage II were partial or total cystectomy[49]. In general status of working and social contact, patients with TURBT might be less affected by the treatment than patients treated by cystectomy. We speculated that perceived support form others (e.g., colleagues and relatives) by patients with cystectomy might offer a sense of hope and confidence, resulting in greater chance to connect with others and engage in social activities, which might further relieve psychological disorders.

After controlling for perceived social support, positive psychological variables also accounted for an additional proportion of variance to depression (17.9%), anxiety (14.5%) and PTSD (14.7%). Optimism and resilience were the independent factors of psychological disorders, which partially supported our previous studies [23] and suggested the protective effects of optimism and resilience in cancer patients. Optimism and resilience as a whole might alleviate psychological disorders by enabling patients to expect positive outcomes and maintain the efforts to attain the health-related goals [26,27], and to mobilize the individual’s resources to successfully maintain or recover their mental health in the face of bladder/kidney cancer [28,29]. Especially among bladder cancer patients with stage II, optimsim was the only internal resource of anxiety and PTSD, indicating that optimism might trump the impacts of resilience on anxiety disorders (Tables E and G in S1 Appendix). Although the causal inference could not be drawn from the cross-sectional design, both positive expectancies regarding favorable outcomes in one’s own future (optimism) and positive adaptation in the context of traumatic events (resilience) were important to successfully ameliorate depression, anxiety and PTSD in bladder/kidney cancer patients.

An interesting and important finding was that when positive psychological variables entered into regression model together, hope represented the significant association with PTSD only, which might be mainly caused by Snyder’s theory of hope that interacted with PTSD and the difference among psychological disorders. PTSD refers to the psychological symptoms (e.g., intrusive cognitions, avoidance and hyperarousal) caused exclusively by the experience of cancer diagnosis and treatment [10], which is different from depression and anxiety (or more precisely of generalized anxiety disorder) that mainly reflect a variety of emotional states, such as unpleasant, worried, negative and hopeless, about individuals’ lives, the world and the future, without reference to specific events [9,23]. Additionally, in Snyder’s theory, hope was considered as goal-directed or problem-directed thinking, including planning to meet goals (pathways) and goal directed energy (agency) [24]. Cancer patients with high level of hope might be able to find solutions to issues caused by cancer and feel confident that they can utilize pathways to solve or cope with those issues. Therefore, hope showed significantly special and targeted association with PTSD, which might provide valuable insight into developing targeted psychotherapy toward cancer patients’ PTSD based on Snyder’s theory of hope.

### Implication

There are several implications in our study. In theory, a whole new perspective would be provided for researchers to build an integrated model to combat psychological disorders in cancer patients by including both perceived social support and positive psychological resources. Furthermore, hope in Snyder’s theory might be an important resource to alleviate PTSD in patients with bladder/kidney cancer [24]. In practice, the high prevalence of depression, anxiety and PTSD in newly diagnosed bladder/kidney cancer patients should receive sufficient attention in Chinese medical settings. The findings in our study also suggest that comprehensive care, including social support from family and psychological resources, is indispensable for bladder/kidney cancer patients. For instance, Cicero et al. pointed out that family members might feel ambivalence towards the cancer patients and engage in unhelpful defenses such as withdrawal and criticism [39]. Therefore, doctors should make clear statement about patients’ illness and patiently communicate with patients and their families. Family members should try to provide reassurance and spend time with patients rather than avoidance and withdrawal. On the other hand, previous studies indicated that hope, optimism and resilience can be enhanced through specific psychotherapy interventions [23,50–52]. As described in detail previously [53], the psychosocial interventions should improve optimism by considering the patients’ situation- or treatment-specific expectations. As introduced in a recent review [50], stress management, resilience training and interventions (e.g., meaning-making interventions) that targeted other aspects of the cancer experience might directly or indirectly improve resilience [51]. Based on Snyder’s theory of hope and the findings of previous studies, Berendes et al. developed a psychosocial intervention module which includes the following key components, 1) discussion on patients’ objective understanding of their disease, 2) recognition of goals and ordering of importance of goals, 3) recognition of realistic short- and long-term goals achievable within the situation of bladder/kidney cancer, 4) confirmation of all the potential pathways toward goals and choosing the most likely pathway to success, and 5) measures to boost agency and monitor pathways [40,52].

### Limitation

Our study had several limitations. First, we used a convenience sample from one oncology center, so generalization from the present results should be attempted with caution. Second, depression, anxiety and PTSD measured by the self-report questionnaires mainly referred to depressive symptom, anxiety symptom and PTSD symptoms in this study. Third, further studies need to be conducted to examine whether the results of the present study are suitable to the different cultural context and other cancer samples. Fourth, besides perceived social support, hope, optimism and resilience, internal and external resources related to psychological disorders should be further explored. Finally, this study was based on cross-sectional design. Thus, causality cannot be implied from the results. Further longitudinal studies are needed to validate the current findings.

## Conclusions

The present study found the high prevalence of depression, anxiety and PTSD among newly diagnosed patients with early-stage bladder/kidney cancer and identified the associated psychosocial factors. Particularly, patient’s perception of social support from family was the only external resource of psychological disorders. Optimism and resilience when to be assessed together showed integrated and independent effects on psychological disorders while hope showed significantly special and targeted association with PTSD. The findings provides a whole new perspective for future research building an integrated model by synthesizing the associated psychosocial resources in cancer patients so as to advocate the role of the integrated psychosocial interventions based on our findings in oncology field.

## Supporting Information

S1 Appendix Supplementary tables. (DOC)

S2 Appendix The raw data of the present study. (XLS)
