# Supplementary material for: Psychological Disorders and Psychosocial Resources of Patients with Newly Diagnosed Bladder and Kidney Cancer: A Cross-Sectional Study
Source: PLoS One. 2016 May 18;11(5):e0155607. doi: 10.1371/journal.pone.0155607 (PMC4871582; doi:10.1371/journal.pone.0155607)
Supplement: S1 Appendix — (DOC) [file pone.0155607.s001.doc]

**Table A** **Study variables scores in bladder and kidney cancer** patients

| **Variables** | **AHS** | **LOT-R** | **RS-14** | **Total-MSPSS** | **MSPSS-others** | **MSPSS-family** | **MSPSS-friend** |
| --- | --- | --- | --- | --- | --- | --- | --- |
| **Cancer type** | *p*=0.077 | *p*=0.795 | *p*=0.725 | *p*=0.033 | *p*=0.099 | *p*=0.087 | *p*=0.005 |
| Bladder | 21.28±4.61 | 10.12±2.09 | 64.62±17.42 | 56.75±17.21 | 18.94±6.07 | 19.63±6.35 | 18.17±5.67 |
| Kidney | 21.99±4.28 | 10.07±2.05 | 65.17±16.83 | 60.08±17.22 | 19.85±6.13 | 20.61±6.16 | 19.62±5.73 |

MSPSS = Multidimensional Scale of Perceived Social Support; AHS = Adult Hope Scale; LOT-R = Life Orientation Scale-Revised; RS-14 = 14-items version of Resilience Scale

Independent sample *t*-test was used.

**Table B Study variables scores in bladder cancer patients with stage I or stage II**

| **Variables** | **N** | **CES-D** | **SAS** | **PCL-C** | **AHS** | **LOT-R** | **RS-14** | **Total-MSPSS** | **MSPSS-others** | **MSPSS-family** | **MSPSS-friend** |
| --- | --- | --- | --- | --- | --- | --- | --- | --- | --- | --- | --- |
| **Stage** |  | *p*=0.161 | *p*=0.497 | *p*=0.088 | *p*=0.210 | *p*=0.169 | *p*=0.266 | *p*=0.033 | *p*=0.066 | *p*=0.025 | *p*=0.045 |
| I | 158 | 22.64±9.31 | 43.62±9.84 | 37.70±12.86 | 21.56±4.66 | 10.26±2.13 | 65.54±18.03 | 58.49±17.32 | 19.47±6.11 | 20.31±6.32 | 18.71±5.75 |
| II | 86 | 24.41±9.54 | 44.50±9.27 | 40.74±13.93 | 20.78±4.49 | 9.87±2.00 | 62.94±16.24 | 53.57±16.64 | 17.98±5.90 | 18.41±6.27 | 17.19±5.44 |

CES-D = Center for Epidemiologic Studies Depression Scale; SAS = Zung Self-Rating Anxiety Scale; PCL-C = Posttraumatic Stress Disorder Checklist-Civilian Version; MSPSS = Multidimensional Scale of Perceived Social Support; AHS = Adult Hope Scale; LOT-R = Life Orientation Scale-Revised; RS-14 = 14-items version of Resilience Scale

Independent sample *t*-test was used.

**Table C Study variables scores in bladder cancer patients with different surgical methods**

| **Variables** | **N** | **CES-D** | **SAS** | **PCL-C** | **AHS** | **LOT-R** | **RS-14** | **Total-MSPSS** | **MSPSS-others** | **MSPSS-family** | **MSPSS-friend** |
| --- | --- | --- | --- | --- | --- | --- | --- | --- | --- | --- | --- |
| **Treatment** |  | *p*=0.993 | *p*=0.988 | *p*=0.870 | *p*=0.776 | *p*=0.576 | *p*=0.733 | *p*=0.518 | *p*=0.598 | *p*=0.363 | *p*=0.524 |
| TURBT | 155 | 23.47±9.24 | 44.05±9.98 | 38.79±13.33 | 21.37±4.89 | 10.23±2.22 | 63.87±18.08 | 57.12±17.45 | 18.95±6.19 | 19.81±6.48 | 18.35±5.63 |
| Partial cystectomy | 48 | 23.42±9.88 | 44.08±9.33 | 39.33±13.95 | 20.58±4.22 | 9.94±1.92 | 63.60±16.96 | 53.81±18.05 | 18.04±6.18 | 18.33±6.54 | 17.43±6.04 |
| Radical cystectomy | 31 | 23.26±9.72 | 43.77±9.07 | 40.13±12.51 | 20.83±4.18 | 9.90±1.78 | 66.45±15.37 | 56.58±15.99 | 19.29±5.72 | 19.84±5.77 | 17.45±5.79 |

TURBT = transurethral resection of bladder cancer; CES-D = Center for Epidemiologic Studies Depression Scale; SAS = Zung Self-Rating Anxiety Scale; PCL-C = Posttraumatic Stress Disorder Checklist-Civilian Version; MSPSS = Multidimensional Scale of Perceived Social Support; AHS = Adult Hope Scale; LOT-R = Life Orientation Scale-Revised; RS-14 = 14-items version of Resilience Scale

One way analysis of variance (ANOVA) was used.

**Table D Study variables scores in bladder cancer patients with newly diagnosed or recurrent cases**

| **Variables** | **N** | **CES-D** | **SAS** | **PCL-C** | **AHS** | **LOT-R** | **RS-14** | **Total-MSPSS** | **MSPSS-others** | **MSPSS-family** | **MSPSS-friend** |
| --- | --- | --- | --- | --- | --- | --- | --- | --- | --- | --- | --- |
| **Recurrent** |  | *p*=0.267 | *p*=0.350 | *p*=0.936 | *p*=0.399 | *p*=0.219 | *p*=0.779 | *p*=0.877 | *p*=0.663 | *p*=0.808 | *p*=0.782 |
| No | 233 | 23.41±9.41 | 44.05±9.59 | 38.76±13.31 | 21.34±4.51 | 10.16±2.08 | 64.69±17.58 | 56.72±17.23 | 18.91±6.09 | 19.66±6.36 | 18.15±5.69 |
| Yes | 11 | 20.18±9.29 | 41.27±10.72 | 39.09±13.66 | 20.14±6.46 | 9.36±2.25 | 63.18±14.46 | 57.55±17.69 | 19.73±5.76 | 19.18±6.57 | 18.64±5.66 |

CES-D = Center for Epidemiologic Studies Depression Scale; SAS = Zung Self-Rating Anxiety Scale; PCL-C = Posttraumatic Stress Disorder Checklist-Civilian Version; MSPSS = Multidimensional Scale of Perceived Social Support; AHS = Adult Hope Scale; LOT-R = Life Orientation Scale-Revised; RS-14 = 14-items version of Resilience Scale

Independent sample *t*-test was used.

**Table E Hierarchical regression analyses for psychosocial resources predicting depression in bladder cancer patients with stage** **I or stage** **II**

| **Variables** | **Step 1(β)** | |  | **Step 2(β)** | | | |  | **Step 3(β)** | | | |
| --- | --- | --- | --- | --- | --- | --- | --- | --- | --- | --- | --- | --- |
|  | **I** | **II** |  | **Model 1,I** | **Model 1,II** | **Model 2,I** | **Model 2, II** |  | **Model 1, I** | **Model 1,II** | **Model 2,I** | **Model 2, II** |
| **Covariates** |  |  |  |  |  |  |  |  |  |  |  |  |
| Age | 0.124 | -0.076 |  | 0.110 | -0.022 | 0.132 | -0.028 |  | 0.106 | 0.005 | 0.116 | -0.001 |
| Gender | -0.003 | -0.071 |  | 0.030 | -0.127 | 0.025 | -0.137 |  | 0.017 | -0.023 | 0.019 | -0.033 |
| Education1 | 0.205* | 0.015 |  | 0.146 | -0.111 | 0.141 | -0.098 |  | 0.104 | -0.108 | 0.101 | -0.099 |
| Education2 | 0.192** | 0.086 |  | 0.152 | 0.029 | 0.157 | 0.033 |  | 0.027 | -0.015 | 0.033 | -0.010 |
| Time since diagnosis | 0.076 | -0.270* |  | 0.111 | -0.127 | 0.109 | -0.133 |  | 0.122 | -0.034 | 0.124 | -0.042 |
| **Predictors (external)** |  |  |  |  |  |  |  |  |  |  |  |  |
| Perceived social support |  |  |  | -0.360*** | -0.428*** |  |  |  | -0.104 | -0.217 |  |  |
| Perceived social support-others |  |  |  |  |  | - | -0.448*** |  |  |  | - | -0.228 |
| Perceived social support-family |  |  |  |  |  | -0.392*** | - |  |  |  | -0.159* | - |
| Perceived social support-friend |  |  |  |  |  | - | - |  |  |  | - | - |
| **Predictors (internal)** |  |  |  |  |  |  |  |  |  |  |  |  |
| Hope |  |  |  |  |  |  |  |  | -0.007 | -0.084 | 0.014 | -0.084 |
| Optimism |  |  |  |  |  |  |  |  | -0.400*** | -0.246* | -0.392*** | -0.231* |
| Resilience |  |  |  |  |  |  |  |  | -0.239** | -0.287* | -0.236** | -0.281* |
| **F** | 1.455 | 1.620 |  | 5.168*** | 4.086** | 6.079*** | 4.575*** |  | 10.551*** | 5.608*** | 11.020*** | 5.691*** |
| **R^2^** | 0.046 | 0.092 |  | 0.170 | 0.237 | 0.195 | 0.258 |  | 0.391 | 0.399 | 0.401 | 0.403 |
| **Adj.R^2^** | 0.014 | 0.035 |  | 0.137 | 0.179 | 0.163 | 0.202 |  | 0.354 | 0.328 | 0.365 | 0.332 |
| **R^2^-changes** | 0.046 | 0.092 |  | 0.125 | 0.145 | 0.149 | 0.166 |  | 0.220 | 0.162 | 0.207 | 0.145 |

β = standardized regression coefficient; Education1 = Middle school or below vs. Junior college or above; Education2 = High school vs. Junior college or above; Adj.R^2^ = adjusted R^2^.

* *p* < 0.05, ** *p* < 0.01, *** *p* < 0.001

There were two models (Model 1 and Model 2) in Step 2. Total score of social support was added in Model 1, and three subscales (i.e., others, friend and family) were added in Model 2 adjusted by the stepwise regression due to the high correlations among the MSPSS subscales

**Table F Hierarchical regression analyses for psychosocial resources predicting anixety in bladder cancer patients with stage** **I or stage** **II**

| **Variables** | **Step 1(β)** | |  | **Step 2(β)** | | | |  | **Step 3(β)** | | | | |
| --- | --- | --- | --- | --- | --- | --- | --- | --- | --- | --- | --- | --- | --- |
|  | **I** | **II** |  | **Model 1,I** | **Model 1,II** | **Model 2,I** | **Model 2, II** |  | **Model 1, I** | **Model 1,II** | **Model 2,I** | **Model 2, II** | |
| **Covariates** |  |  |  |  |  |  |  |  |  |  |  |  |  |
| Age | 0.084 | -0.123 |  | 0.068 | -0.064 | 0.093 | -0.071 |  | 0.055 | -0.038 | 0.068 | -0.045 |  |
| Gender | -0.052 | -0.076 |  | -0.014 | -0.137 | -0.020 | -0.147 |  | -0.030 | -0.032 | -0.028 | -0.043 |  |
| Education1 | 0.138 | -0.091 |  | 0.071 | -0.227 | 0.065 | -0.211 |  | 0.025 | -0.229 | 0.020 | -0.216 |  |
| Education2 | 0.182 | 0.020 |  | 0.137 | -0.041 | 0.142 | -0.036 |  | 0.019 | -0.086 | 0.026 | -0.079 |  |
| Time since diagnosis | 0.025 | -0.180 |  | 0.064 | -0.026 | 0.063 | -0.035 |  | 0.072 | 0.065 | 0.074 | 0.055 |  |
| **Predictors (external)** |  |  |  |  |  |  |  |  |  |  |  |  |  |
| Perceived social support |  |  |  | -0.412*** | -0.460*** |  |  |  | -0.161* | -0.261* |  |  |  |
| Perceived social support-others |  |  |  |  |  | - | -0.474*** |  |  |  | - | -0.265* |  |
| Perceived social support-family |  |  |  |  |  | -0.451*** | - |  |  |  | -0.223** | - |  |
| Perceived social support-friend |  |  |  |  |  | - | - |  |  |  | - | - |  |
| **Predictors (internal)** |  |  |  |  |  |  |  |  |  |  |  |  |  |
| Hope |  |  |  |  |  |  |  |  | -0.100 | -0.099 | -0.073 | -0.098 |  |
| Optimism |  |  |  |  |  |  |  |  | -0.311*** | -0.243* | -0.301*** | -0.226* |  |
| Resilience |  |  |  |  |  |  |  |  | -0.206* | -0.259 | -0.205* | -0.257 |  |
| **F** | 0.923 | 1.175 |  | 6.020*** | 4.059** | 7.390*** | 4.490** |  | 9.927*** | 5.376*** | 10.678*** | 5.425*** |  |
| **R^2^** | 0.029 | 0.068 |  | 0.193 | 0.236 | 0.227 | 0.254 |  | 0.376 | 0.389 | 0.394 | 0.391 |  |
| **Adj.R^2^** | -0.002 | 0.010 |  | 0.161 | 0.178 | 0.196 | 0.198 |  | 0.339 | 0.317 | 0.357 | 0.319 |  |
| **R^2^-changes** | 0.029 | 0.068 |  | 0.164 | 0.167 | 0.198 | 0.186 |  | 0.183 | 0.153 | 0.167 | 0.137 |  |

β = standardized regression coefficient; Education1 = Middle school or below vs. Junior college or above; Education2 = High school vs. Junior college or above; Adj.R^2^ = adjusted R^2^.

* *p* < 0.05, ** *p* < 0.01, *** *p* < 0.001

There were two models (Model 1 and Model 2) in Step 2. Total score of social support was added in Model 1, and three subscales (i.e., others, friend and family) were added in Model 2 adjusted by the stepwise regression due to the high correlations among the MSPSS subscales

**Table G Hierarchical regression analyses for psychosocial resources predicting PTSD in bladder cancer patients with stage** **I or stage** **II**

| **Variables** | **Step 1(β)** | |  | **Step 2(β)** | | | |  | **Step 3(β)** | | | |
| --- | --- | --- | --- | --- | --- | --- | --- | --- | --- | --- | --- | --- |
|  | **I** | **II** |  | **Model 1,I** | **Model 1,II** | **Model 2,I** | **Model 2, II** |  | **Model 1, I** | **Model 1,II** | **Model 2,I** | **Model 2, II** |
| **Covariates** |  |  |  |  |  |  |  |  |  |  |  |  |
| Age | 0.075 | -0.066 |  | 0.060 | -0.027 | 0.084 | -0.030 |  | 0.051 | -0.003 | 0.063 | -0.004 |
| Gender | -0.047 | -0.054 |  | -0.012 | -0.093 | -0.017 | -0.103 |  | -0.025 | 0.019 | -0.024 | 0.013 |
| Education1 | 0.179 | -0.061 |  | 0.116 | -0.151 | 0.110 | -0.145 |  | 0.072 | -0.163 | 0.068 | -0.162 |
| Education2 | 0.248* | -0.013 |  | 0.206 | -0.053 | 0.211* | -0.052 |  | 0.091 | -0.100 | 0.097 | -0.099 |
| Time since diagnosis | 0.025 | -0.259* |  | 0.062 | -0.158 | 0.060 | -0.158 |  | 0.072 | -0.051 | 0.074 | -0.053 |
| **Predictors (external)** |  |  |  |  |  |  |  |  |  |  |  |  |
| Perceived social support |  |  |  | -0.386*** | -0.303* |  |  |  | -0.139 | -0.090 |  |  |
| Perceived social support-others |  |  |  |  |  | - | -0.330** |  |  |  | - | -0.107 |
| Perceived social support-family |  |  |  |  |  | -0.422*** | - |  |  |  | -0.198* | - |
| Perceived social support-friend |  |  |  |  |  | - | - |  |  |  | - | - |
| **Predictors (internal)** |  |  |  |  |  |  |  |  |  |  |  |  |
| Hope |  |  |  |  |  |  |  |  | -0.031 | -0.184 | -0.006 | -0.185 |
| Optimism |  |  |  |  |  |  |  |  | -0.336*** | -0.230* | -0.327*** | -0.222* |
| Resilience |  |  |  |  |  |  |  |  | -0.245** | -0.259 | -0.243** | -0.250 |
| **F** | 1.481 | 1.419 |  | 5.906*** | 2.401* | 7.043*** | 2.726* |  | 9.987*** | 4.519*** | 10.618*** | 4.559*** |
| **R^2^** | 0.046 | 0.081 |  | 0.190 | 0.154 | 0.219 | 0.172 |  | 0.378 | 0.349 | 0.392 | 0.351 |
| **Adj.R^2^** | 0.015 | 0.024 |  | 0.158 | 0.090 | 0.188 | 0.109 |  | 0.340 | 0.271 | 0.355 | 0.274 |
| **R^2^-changes** | 0.046 | 0.081 |  | 0.144 | 0.073 | 0.172 | 0.090 |  | 0.188 | 0.194 | 0.174 | 0.179 |

β = standardized regression coefficient; Education1 = Middle school or below vs. Junior college or above; Education2 = High school vs. Junior college or above; Adj.R^2^ = adjusted R^2^.

* *p* < 0.05, ** *p* < 0.01, *** *p* < 0.001

There were two models (Model 1 and Model 2) in Step 2. Total score of social support was added in Model 1, and three subscales (i.e., others, friend and family) were added in Model 2 adjusted by the stepwise regression due to the high correlations among the MSPSS subscales
